# 

**Published:** 1892-10-08

**Authors:** 


### CONTENTS.

**A Plea for the "Practical" Man** - - - - - - - 17

**Passing Topics.**—The Cost of Trained Nurses - - - - 18

**Diet in Disease.** By Mrs. Ernest Hart. II.—Food and Food Values - - - - - - - - - - 19

**Everybody's Page** - - - - - - - - - - 20

**Medical History from the Earliest Times.** By E. T. Withington, M.A., M.B., Oxon. XXVI.—The Eclectics and Compilers - - - - - - - - - - 21

**Employments for Women.**—IV. Lecturing for the County Councils - - - - - - - - - - 22

**Annotations.**—Cholera One Step Further—Aid to the Injured—The "Pall Mall Gazette" inside the Hospitals - - - - 23

**Notes and News** - - - - - - - - - - 24

**Scraps and Gleanings** - - - - - - - - - 32

**The Hospital Clinic—**

St. Bartholomew's Hospital.—The Treatment of Chronic Constipation - - - - - - - - - - - - 25

Bath Royal Mineral Water Hospital.—Treatment of Rheumatoid Arthritis - - - - - - - - - - 26

**New Drugs, Appliances, and Things Medical**—Messrs. Fry and Sons' Cocoa and Chocolate—Toilet Specialities - - 28

**The Institutional Workshop—**

Within the Hospitals.—Aberdeen and its Institutions - 29

Hospital Administration. IV.—Special Hospitals—Lying-in and Women - - - - - - - - - - 31

Hospital Construction.—The New Isolated Building at the "Oresund Hospital," Copenhagen - - - - - - - 32

**The Student of Medicine,**—Appointments—Vacancies

### EXTRA SUPPLEMENT.—THE NURSING MIRROR.

**En Passant.**—News from Nicosia—Brabazon Home of Comfort, Reigate—Midleton Guardians and the Nuns—Children's Hospitals and Convalescent Homes—Tottenham Temporary Fever Hospital—Nurses in Workhouses—An Ungrateful Convalescent—Tyn-y-coed Convalescent Home—An Out-Patient Department - - - vii

**Lectures for Asylum Attendants.** By William Harding, M.B. IV.—Warmth - - - - - - - - - - viii

**St. Luke's Home, Vancouver.**—One Hundred Pounds Wanted - ix

**For Reading to the Sick.**—Our Pattern - - - - - - ix

**Nursing Homes.**—VI. Guy's Hospital - - - - - - x

**Nurses' Bookshelf** - - - - - - - - - - x

**Home for the Dying, Friedenheim** - - - - - - xi

**Everybody's Opinion.**—Nurses' Co-operation—Metropolitan District Schools—The Worcester Infirmary - - - - - xi

**Appointments—Where to Go** - - - - - - - xi

**Notes and Queries—Wants and Workers** - - - - xi

**Off Duty.**—"The Result of a Chill"—**Hints for Nurses** - - xii

